# Optic nerve head and retinal blood flow regulation during isometric exercise as assessed with laser speckle flowgraphy

**DOI:** 10.1371/journal.pone.0184772

**Published:** 2017-09-12

**Authors:** Katarzyna J. Witkowska, Ahmed M. Bata, Giacomo Calzetti, Nikolaus Luft, Klemens Fondi, Piotr A. Wozniak, Doreen Schmidl, Matthias Bolz, Alina Popa-Cherecheanu, René M. Werkmeister, Gerhard Garhöfer, Leopold Schmetterer

**Affiliations:** 1 Department of Clinical Pharmacology, Medical University of Vienna, Vienna, Austria; 2 Department of Ophthalmology, University of Parma, Parma, Italy; 3 Department of Ophthalmology, Kepler University Hospital, Linz, Austria; 4 Department of Ophthalmology, Medical University of Warsaw, Warsaw, Poland; 5 Carol Davila University of Medicine and Pharmacy, Bucharest, Romania; 6 Department of Ophthalmology, Emergency University Hospital, Bucharest, Romania; 7 Center for Medical Physics and Biomedical Engineering, Medical University of Vienna, Vienna, Austria; 8 Singapore Eye Research Institute, Singapore, Singapore; 9 Lee Kong Chian School of Medicine, Nanyang Technological University, Singapore, Singapore; University of California San Diego, UNITED STATES

## Abstract

The aim of the present study was to investigate regulation of blood flow (BF) in the optic nerve head (ONH) and a peripapillary region (PPR) during an isometric exercise-induced increase in ocular perfusion pressure (OPP) using laser speckle flowgraphy (LSFG) in healthy subjects. For this purpose, a total of 27 subjects was included in this study. Mean blur rate in tissue (MT) was measured in the ONH and in a PPR as well as relative flow volume (RFV) in retinal arteries (ART) and veins (VEIN) using LSFG. All participants performed isometric exercise for 6 minutes during which MT and mean arterial pressure were measured every minute. From these data OPP and pressure/flow curves were calculated. Isometric exercise increased OPP, MT_ONH_ and MT_PRR_. The relative increase in OPP (78.5 ± 19.8%) was more pronounced than the increase in BF parameters (MT_ONH_: 18.1 ± 7.7%, MT_PRR_: 21.1 ± 8.3%, RFV_ART_: 16.5 ±12.0%, RFV_VEIN_: 17.7 ± 12.4%) indicating for an autoregulatory response of the vasculature. The pressure/flow curves show that MT_ONH_, MT_PRR_, RFV_ART_, RFV_VEIN_ started to increase at OPP levels of 51.2 ± 2.0%, 58.1 ± 2.4%, 45.6 ± 1.9% and 45.6 ± 1.9% above baseline. These data indicate that ONHBF starts to increase at levels of approx. 50% increase in OPP: This is slightly lower than the values we previously reported from LDF data. Signals from the PPR may have input from both, the retina and the choroid, but the relative contribution is unknown. In addition, retinal BF appears to increase at slightly lower OPP values of approximately 45%. LSFG may be used to study ONH autoregulation in diseases such as glaucoma.

***Trial Registration*:** ClinicalTrials.gov NCT02102880

## Introduction

The human optic nerve is a structure with vascular supply from different sources.[1, 2] The nerve fiber layer is nourished by vessels that get their supply from the central retinal artery. The pre-laminar region gets its vascular input from the peripapillary choroid. The lamina cribrosa is nourished by branches from the short posterior ciliary arteries, either directly or from the circle of Zinn-Haller. The retrolaminar region contains vessels that stem from the pial vascular plexus as well as from the axial centrifugal vascular supply.

Alterations in optic nerve head (ONH) blood flow have been implicated in the pathogenesis of glaucoma. More specifically ONH ischemia and altered ONH blood flow autoregulation may play a role in the processes that lead to axon damage and subsequent loss of retinal ganglion cells.[2–5] Indeed data from a variety of studies have provided evidence for altered autoregulation in glaucoma.[6, 7]

Due to the complexity of optic nerve blood supply relatively little is known about regulation of blood flow in this region. To date no technique is capable of measuring all aspects of ONH autoregulation.[8] Most data on the regulation of ONH blood flow arise from studies that use laser Doppler flowmetry (LDF).[9–18] In Japanese subjects laser speckle flowgraphy (LSFG) was employed for studying ONH regulation.[19] We set out to study ONH blood flow regulation during an isometric exercise-induced increase in ocular perfusion pressure (OPP) in healthy white subjects. Data were compared to our previously published data using LDF.

## Methods

### Subjects

The protocol of this prospective study was approved by the Ethics Committee of the Medical University of Vienna and the study was conducted at the Department of Clinical Pharmacology of the Medical University of Vienna. All 27 participating subjects gave written informed consent after the nature and possible consequences of the study had been explained in detail. All subjects finished the study according to the protocol and no dropouts occurred. Study procedures adhered to the guidelines outlined in the Declaration of Helsinki. Subjects were recruited and completed the study between December 2015 and June 2016.

All subjects underwent a screening examination during the two weeks prior to the study day that consisted of medical history, physical examination, and a full ophthalmologic examination including best-corrected visual acuity testing with standard Early Treatment of Diabetic Retinopathy Study (ETDRS) charts, slit-lamp examination including indirect funduscopy and measurement of intraocular pressure (IOP) using Goldmann applanation tonometry. In addition, systolic blood pressure (SBP) and diastolic blood pressure (DBP) were measured with automated oscillometry and a urine pregnancy test was performed in women. According to the study protocol the following exclusion criteria were defined: smoking, ametropia ≥ 6 diopters, contact lens wear, any ocular surgery, and opacities of the optical media (e.g. corneal scars, LOCS-II grading ≥ 3, vitreous opacities) potentially interfering with the measurement procedures. In addition, any other relevant ocular disease or abnormality as well as any clinically relevant illness as judged by the investigators were considered as exclusion. Further exclusion criteria were systemic hypertension, pregnancy or lactation, intake of any medication in the three weeks preceding the study as well as a blood donation in the three weeks prior to the study. In all subjects one eye was randomly selected as the study eye. All subjects abstained from alcohol and stimulating beverages containing xanthine derivatives (tea, coffee, cola-like drinks) for at least 12 hours before the measurements were performed.

### Procedures and interventions

All measurements were performed after a resting period of at least 20 minutes during which subjects remained in a sitting position. Stability of systemic blood pressure and pulse rate was verified by repeated measurements before the actual study procedures were started.

Before isometric exercise was started a baseline measurement using LSFG was performed and systemic blood pressure, pulse rate (PR) and IOP were recorded while subjects were comfortably sitting in a chair. Thereafter, isometric exercise was started and maintained for 6 minutes. This period of isometric exercise consisted of squatting in a position where the upper and the lower legs formed almost a right angle. When the squatting period was started the chair was carefully removed and the subjects were asked to remain in their position, which ensures that the position between the head of the subject and the LSFG instrument does not change. This type of exercise is associated with a pronounced increase in mean arterial blood pressure. Measurement of ONH blood flow, systemic blood pressure and pulse rate was performed every minute throughout these experiments.

### Measurements

Systemic blood pressure and pulse rate: Systolic, diastolic and mean arterial pressures (SBP, DBP, MAP) were measured on the upper arm using an automated oscillometric device. PR was automatically recorded from a finger pulse-oxymetric device.

Intraocular pressure and ocular perfusion pressure: IOP was measured with a Goldmann applanation tonometer mounted on a slit lamp. Oxybuprocainhydrochloride was used for local anesthesia. OPP in the sitting position was calculated as OPP = 2/3*MAP-IOP [20, 21] accounting for the hydrostatic pressure difference between the eye and the upper arm when subjects are seated. In addition, IOP was assumed to equal pressure in ocular veins.

**Fig 1:**
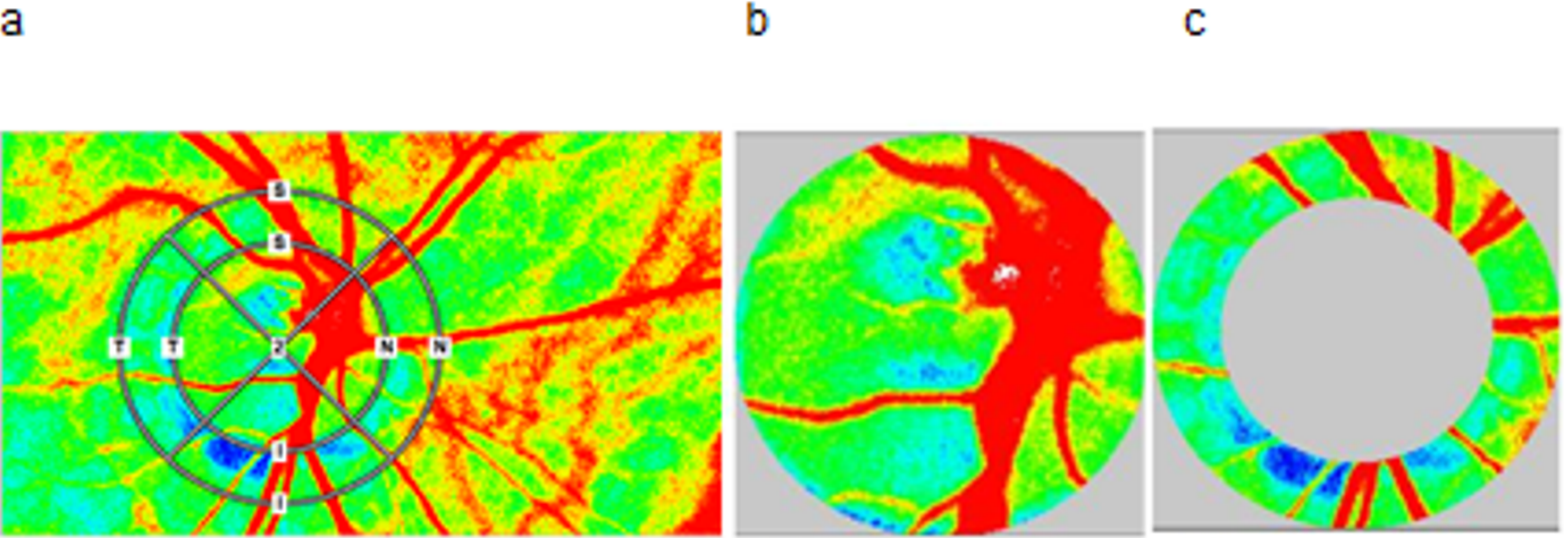
Laser speckle flowgraphy (LSFG) sample measurement of optic nerve head (ONH) and peripapillary region (PPR) mean blur rate in different regions. (a). An inner elliptical band was manually fitted to ONH borders; the obtained area represents the ONH area (b). The correct identification of the borders was optimized by comparing the LSFG image with a fundus photograph. A second outer elliptical band was obtained by increasing the length of both axes of the first ellipse by 50%. The donut-shaped area, obtained after subtraction of ONH area, represents the PPR area (c). For analysis signals from large vessels were not taken into account. In the present case the band was almost circular, but this was not the case in subjects.

Laser Speckle Flowgraphy: In the present study, a commercially available LSFG system (LSFG-NAVI; Softcare Co., Ltd., Fukuoka, Japan) was used. The principles of LSFG were described in detail in recent review papers. [22, 23] Briefly, the LSFG system used in the present study consists of a fundus camera equipped with a single-mode diode laser emitting light at a wavelength of 830 nm and a digital charge-coupled device (CCD) camera with 750 x 360 pixels. The primary outcome parameter as obtained with LSFG is mean blur rate (MBR), which is a measure of relative blood flow velocity and is expressed in arbitrary units (AU). It is calculated based on the speckle pattern produced by interference of the laser light scattered by erythrocytes moving in the ocular blood vessels. A total of 118 images are continuously acquired at a frame rate of 30 Hz. As such the total measurements time is approximately 4 seconds. Data are analyzed by in-built software (LSFG Analyzer, Version 3.1.58; Softcare Co., Ltd.) that synchronizes and averages the captured MBR images obtained during the different cardiac cycles. The outcome is a composite map showing the distribution of blood flow at the posterior pole of the eye. The ONH area is manually delineated by positioning an ellipsoid rubber band at the ONH margin (see Fig 1). To optimize this procedure we used a black-white photo provided by LSFG software to precisely delineate the ONH margin for comparison. In addition, blood flow in a peripapillary region (PPR) area was studied. For this purpose a second outer elliptical band was obtained increasing the length of both axes of the first ellipse by 50%. After subtraction of the ONH area this resulted in donut-shaped area representing a PPR of interest supplied by retinal vessels in the inner retina and by choroidal vessel in the peri-papillary choroid.

**Fig 2:**
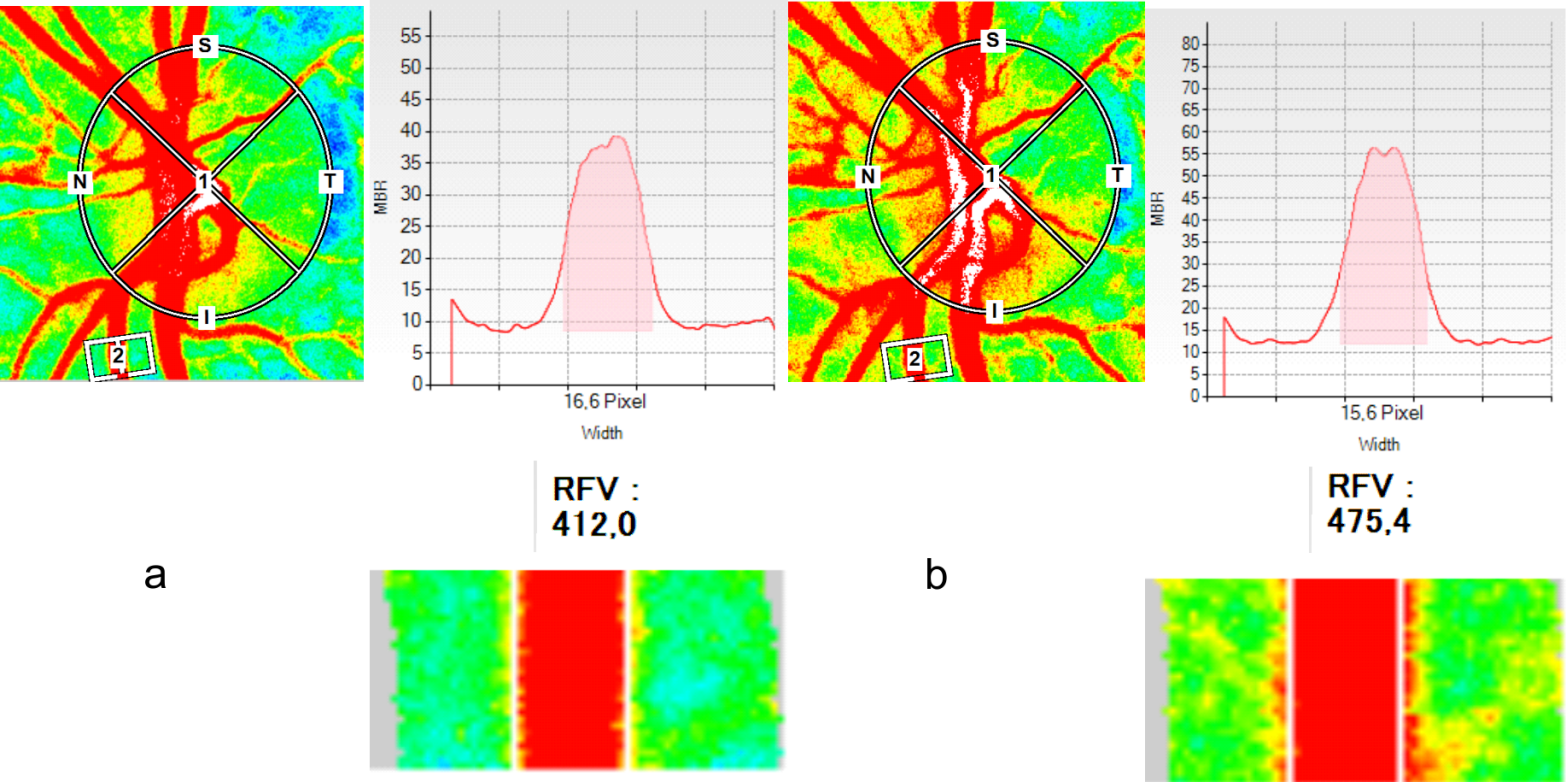
Laser speckle flowgraphy (LSFG) sample measurement in a retinal vessel. Retinal flow volume (RFV) and vessel diameter of a retinal artery segment are evaluated at rest (a) and during isometric exercise (b).

Areas of larger vessels and tissue areas containing microvessels are automatically detected using the LSFG software applying a pre-defined threshold for MBR (vessel extraction function). Thus, MBR can be either determined separately for microvascular areas (MT, “MBR of tissue area”) or for larger vessel (MV, “MBR of vascular area”). In the present study only MT data were analyzed (MT_ONH_, MT_PPR_). For measurements in retinal vessels an approach to quantify blood flow was used.[24, 25] A rectangular band was centered on the retinal vessel of interest. The system is capable of automatically delineating the artery and the vein (Fig 2). The MBR values in the retinal arteries and veins are automatically corrected by the background signal arising from the underlying choroid. The vessel diameter determined by LSFG is given in pixels and used for calculation of relative flow volume (RFV).

The reproducibility of the system has been reported previously. In healthy subjects of Western European descent we reported coefficients of variation between 5.72 and 6.11%.[26] This is in good agreement with previous data in Japanese subjects reporting values between 3 and 11%.[27–30] Reproducibility of RFV was 5.9% and 5,6% in Japanese and white subjects, respectively.

### Data analysis

For data description %-changes from baseline were calculated. A one-way ANOVA model was used to study the time effect of MT and OPP during squatting. In addition, pressure-flow relationships were calculated as described in more details previously.[31–33] Briefly, the relative OPP data were sorted according to ascending values. Given that 27 healthy subjects participated and 6 values were obtained in each subject during isometric exercise this results in a total of 162 OPP/MT values. Data were pooled into 9 groups in the pressure/flow relationship each consisting of 18 individual values. A statistically significant deviation from baseline MT was defined when the 95% confidence interval did not overlap with the baseline value any more. A p-value < 0.05 was considered the level of significance. Statistical analysis was carried out using CSS Statistica for Windows^®^ (Statsoft Inc., Version 6.0, Tulsa, California).

## Results

**Fig 3:**
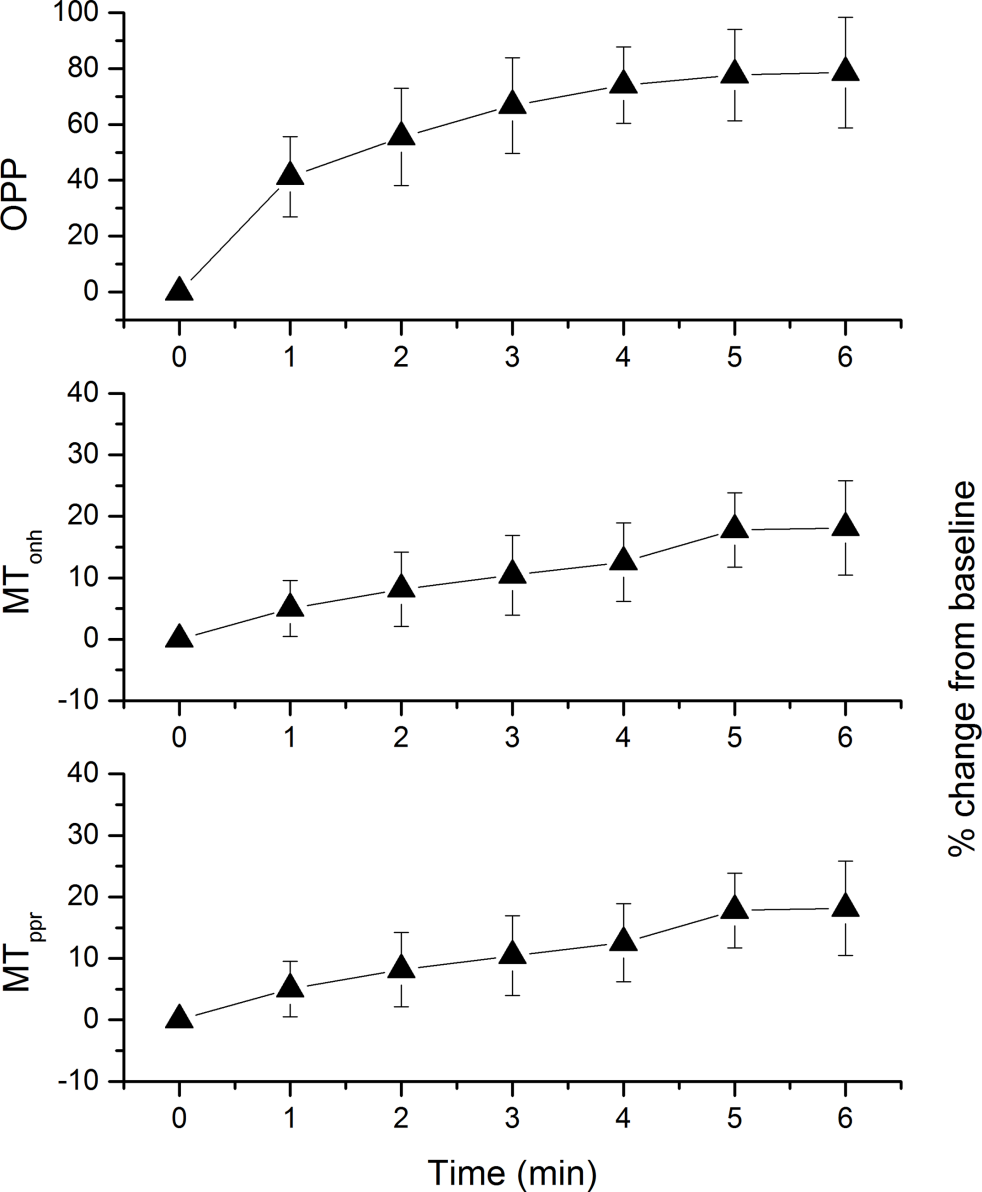
Ocular perfusion pressure (OPP) and mean blur rate (MBR) in tissue area of optic nerve head (MT_ONH_) and peripapillary region (MT_PPR_) during isometric exercise. Data are expressed as % change from baseline. Data are presented as means ± SD (n = 27).

**Fig 4:**
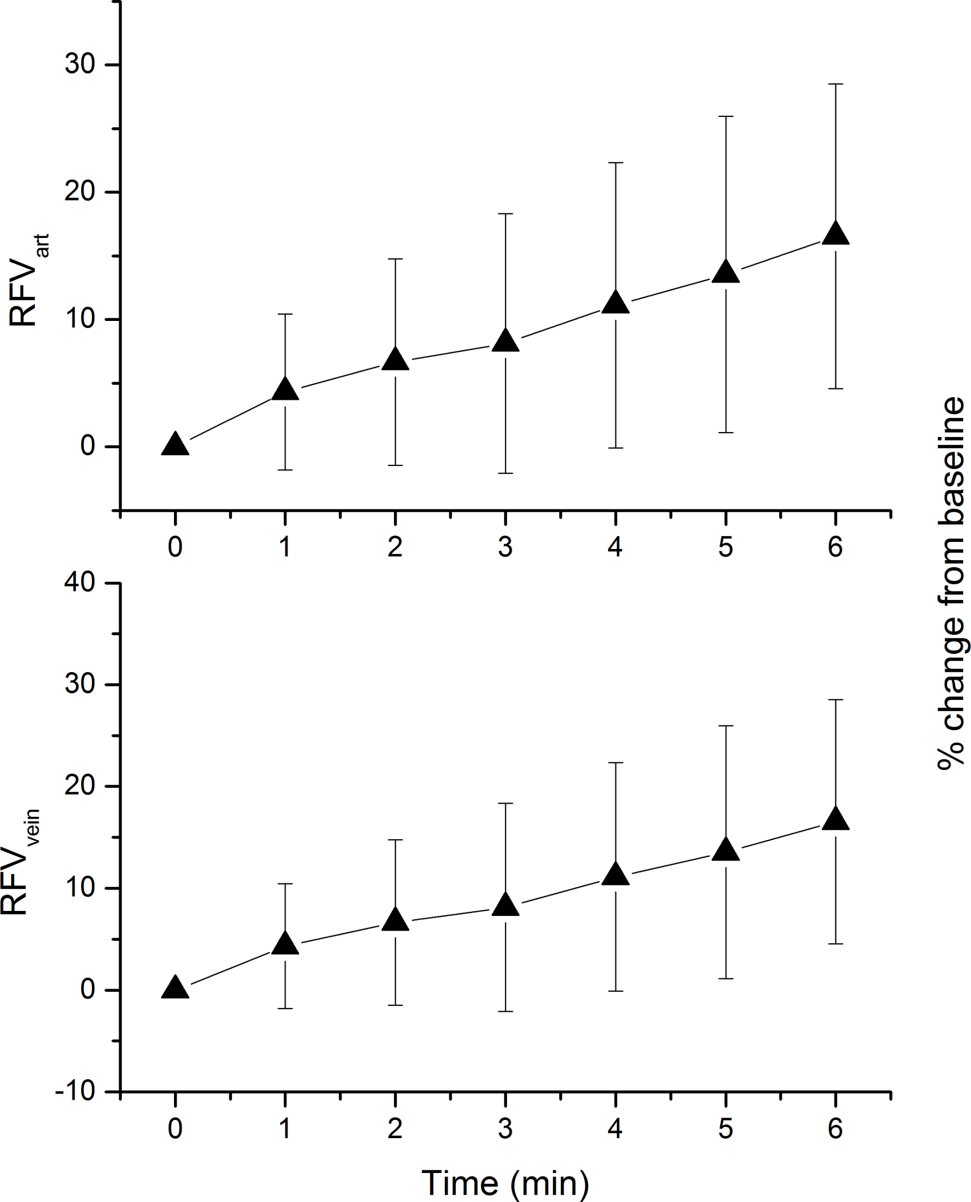
Relative flow volume (RFV) in arteries (RFV_ART_) and veins (RFV_ART_) during isometric exercise. Data are expressed as % change from baseline. Data are presented as means ± SD (n = 27).

27 subjects aged between 18 and 34 years participated in the present study. The baseline data of the participating subjects are presented in Table 1. Isometric exercise-induced changes in MT and OPP are shown in Fig 3. The increase in MT_ONH_, MT_PPR_ and OPP was highly significant (p < 0.001 each). The % change in MT_ONH_, and MT_PPR_ was, however, small as compared to the % change in OPP, which is indicative for blood flow autoregulation. After 6 minutes the relative increase in OPP was 78.5 ± 19.8%, whereas it was only 18.1 ± 7.7% and 21.1 ± 8.3% for MT_ONH_ and MT_PPR_, respectively. The squatting-induced increase in MT_ONH_ and MT_PPR_ was, however, comparable. The change in RFV during isometric exercise is presented in Fig 4. After 6 minutes the relative increase was 16.5 ±12.0% in arteries and 17.7 ± 12.4% in veins. No gender differences were observed in the time course of ocular hemodynamic parameters during isometric exercise (MT_ONH_: p = 0.542, MT_PPR,_: p = 0.617, RFV_ART_: p = 0.788, RFV_VEIN_, p = 0.424). The magnitude of blood flow change in response to the OPP increase had no association with baseline OPP (MT_ONH_: p = 0.471, MT_PPR,_: p = 0.441, RFV_ART_: p = 0.743, RFV_VEIN_, p = 0.663)

**Table 1:** Baseline characteristics of the healthy subjects (n = 27).

Sex (male/female)	11/16
Age (years)	24.6 ± 5.0
Mean arterial pressure (mmHg)	86.7 ± 8.3
Pulse rate (beats/min)	66.0 ± 11.7
Intraocular pressure (mmHg)	15.0 ± 2.4
Ocular perfusion pressure (mmHg)	42.8 ± 6.4
Mean blur rate tissue area_ONH_ (a.u.)	13.7 ± 1.8
Mean blur rate tissue area_PPR_ (a.u.)	12.8 ± 1.9
Retina Flow Volume_ART_ (a.u.)	295.6 ± 61.2
Retina Flow Volume_VEIN_ (a.u.)	387.3 ± 75.2

**Fig 5:**
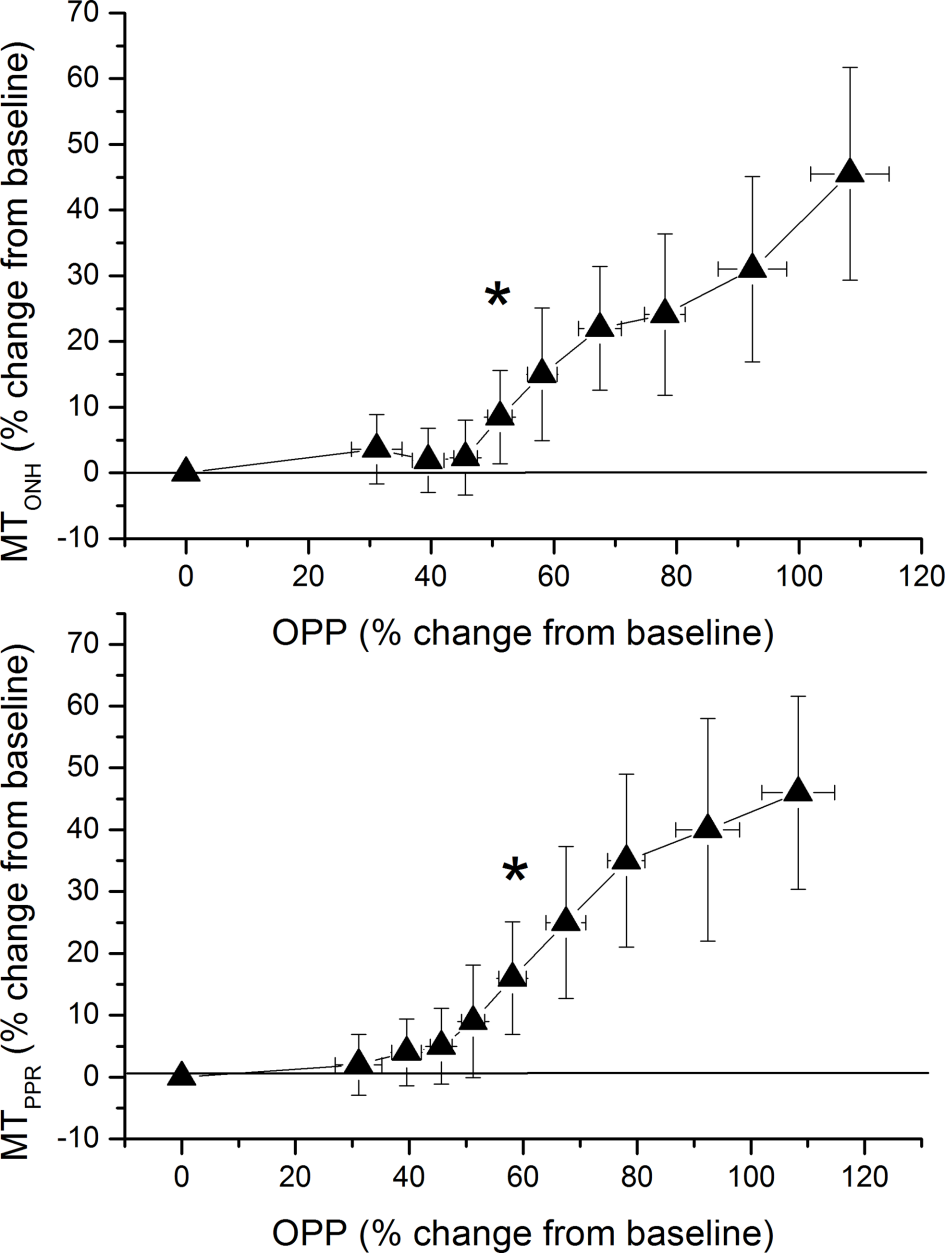
Pressure-flow relationship using the categorized ocular perfusion pressure (OPP)—mean blur rate in tissue area data of optic nerve head (MT_ONH_, upper panel) and peripapillary region (MT_PPR_, lower panel) during isometric exercise. Relative data were sorted into 9 groups consisting of 18 individual values according to ascending OPP values. The means and the 95% confidence intervals are shown (n = 27). Asterisks indicate significant increase from baseline blood flow values.

**Fig 6:**
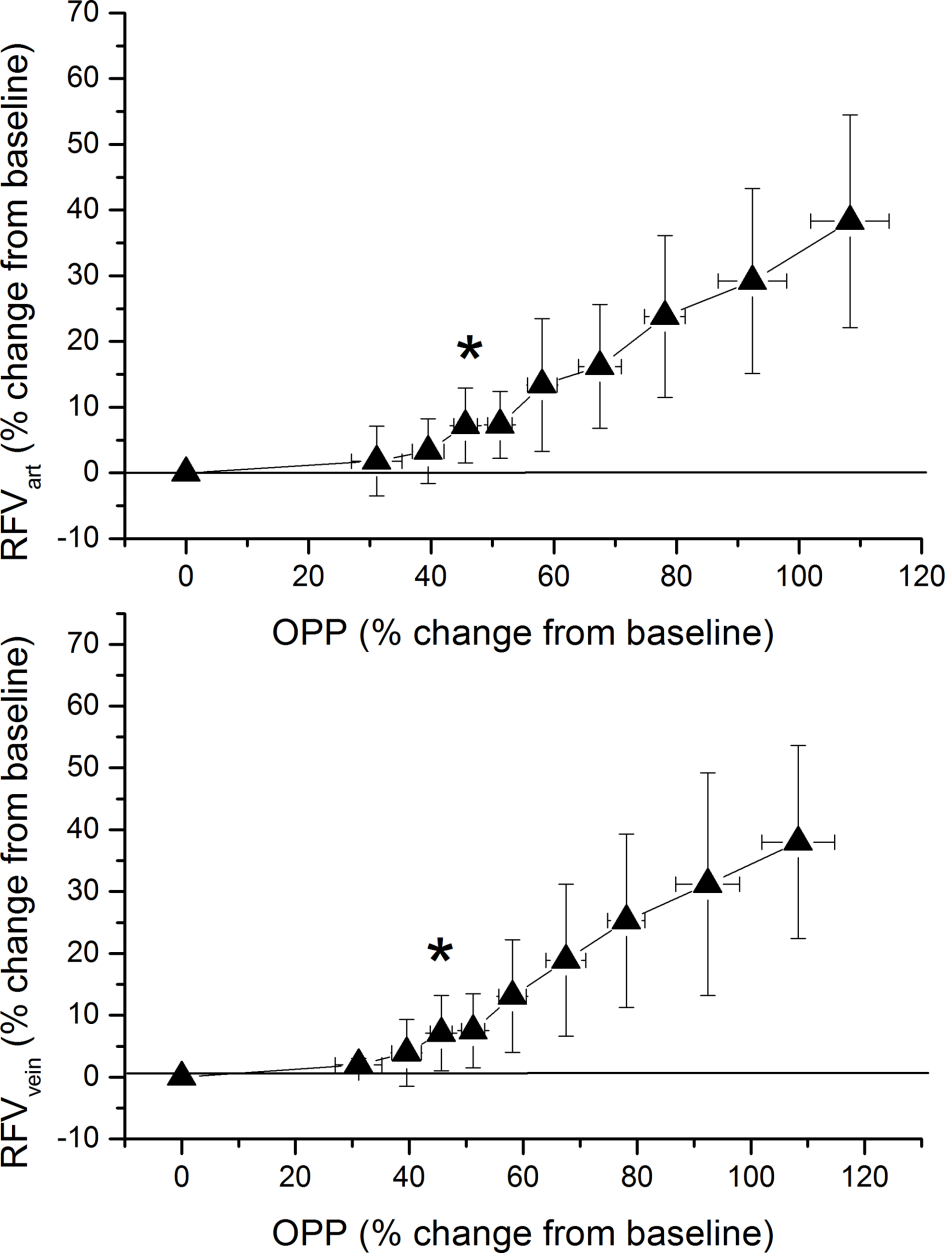
Pressure-flow relationship using the categorized ocular perfusion pressure (OPP)–retinal flow volume in arteries (RFV_ART_, upper panel) and retinal flow volume in veins (RFV_VEIN_, during isometric exercise. Relative data were sorted into 9 groups consisting of 18 individual values according to ascending OPP values. The means and the 95% confidence intervals are shown (n = 27). Asterisks indicate significant increase from baseline blood flow values.

Pressure-flow relationship as calculated from OPP and MT data are presented in Fig 5. For MT_ONH_ a significant increase was seen when OPP levels reached a value of 51.2 ± 2.0% above baseline. For MT_PPR_ the OPP level at which an increase was seen was slightly higher (58.1 ± 2.4%) and this effect was statistically different between the two measurement sites (p = 0.017). Pressure-flow relationship for RFV is presented in Fig 6. For both retinal arteries and retinal veins a significant increase was seen when OPP levels reached a value of 45.6 ± 1.9% above baseline. Of note the graphs presented in Fig 5 and Fig 6 show relative changes over baseline. To depict pressure/flow graphs in humans based on absolute values is not possible, because different subjects start at different baseline blood pressure values.

## Discussion

In the present study we observed that blood flow is autoregulated during isometric exercise both in the ONH and PPR as well as in the retina. Values for upper limit of autoregulation as obtained in the ONH with LSFG are slightly lower than with those obtained using LDF in healthy subjects.[11, 13, 15–17] Using the same protocol for isometric exercise (6 minutes of squatting) previous LDF data indicate that ONH blood flow is effectively autoregulated until OPP increases by approximately 60%, whereas in the present study values of approx. 50% were found.

The principle of LSFG is to image the speckle pattern with an exposure time longer than the shortest speckle fluctuation time.[34] With the LSFG machine used in this study the blur of the speckle, reflecting a reduction in the local speckle contrast, is quantified. As such the technique shares many similarities with LDF, but is capable of producing a two-dimensional map without scanning of the laser beam. Goodman has shown [35] that a relationship exists between the variance of a time-averaged moving speckle pattern and the temporal fluctuation statistics. Whereas LSFG measures the former LDF measures the latter by quantifying autocovariance. If the density of red blood cells is low, the first moment of the power spectrum scales linearly with velocity and concentration (a parameters termed Volume in LDF research). Based on the theory of Bonner and Nossal[36] this concept can be generalized also for tissues containing higher concentrations of red blood cells given that some assumptions are fulfilled. This forms the basis for blood perfusion measurements in arbitrary units using Doppler technology.[37, 38] With LSFG it is not entirely clear whether velocity or flow is measured.[34] The loss of contrast in a speckle pattern will obviously depend on the relation between static and moving scatterers in the sampling volume.[39, 40] Moreover, the velocity distribution will have an impact on the contrast in the speckle pattern. In vivo a combination between Gaussian velocity distribution and Lorentzian distribution may be the most appropriate to describe this dependence.[41] As such it is clear that MT is neither directly related to velocity nor to flow, but the association may depend on the velocity distribution as well as the fraction of moving particles.

Whereas most papers dealing with LSFG in the eye claim that the velocity is measured comparison has been done mainly with technology that measures perfusion. Indeed, LSFG has been validated for measurement of ONH blood flow using hydrogen clearance[27, 42, 43] or fluorescence microspheres[44] as reference method. It is also not fully established from which depth the LSFG signal in the ONH or the PRR arises.[45] It has been shown that the signal from retinal locations exepct the fovea contains retinal as well as choroidal contributions.[22, 23] In this respect it is important to mention that the results obtained during isometric exercise in the PPR showed some similarities to those obtained from the foveal region using LDF where only the subfoveal choroidal vessels contribute to the signal.[9, 46, 47] Very few studies have looked into the proportion of signal arising from retina and choroid, but a LDF study using 100% oxygen breathing as stimulus indicates that the signal arises primarily from the choroid.[48] The present study may indicate that this also holds true for LSFG. Non-human primate data indicate that LDF in the ONH measures preferentially the superficial layers supplied from the central retina artery.[45] On the other hand the large choroidal contribution to the signal obtained from the peripheral retina indicates that deeper structures may contribute to the LSFG signal in the ONH as well. It can also not fully be excluded that MT_ONH_ and/or MT_PPR_ show a zero-setoff although we deem this unlikely based on the measurement principle. All these effects may explain to a certain degree the small differences as observed between LSFG and LDF values during isometric exercise.

Measurement of RFV to quantify blood flow in the retinal vasculature based on LSFG is a relatively new approach.[24] Previous studies have proven adequate reproducibility but some problems with validity of the technique have been reported.[24, 25] Whereas comparison with both laser Doppler velocimetry (LDV) and Doppler OCT revealed significant correlations a significant zero-setoff was observed. This may be a significant problem when studying absolute blood flow values, but less a problem when retinal blood flow changes in a relatively small range. In the present study we observed relatively consistent results between retinal arteries and veins supporting this assumption, Interestingly studies reporting isometric exercise-induced changes in retinal blood flow are sparse. A study using the blue-field entoptic technique observed that white blood cell flux increased at OPP levels between 35% and 42% above baseline.[49] This is in good agreement with laser Doppler velocimetry studies reporting an increase in retinal blood flow at OPP values of 40% above baseline.[20]

Only few studies have so far used LSFG to study autoregulation of ocular blood flow during a change in blood pressure in humans. In response to changes in posture differences between choroidal and ONH blood flow were reported.[29] During both isometric and dynamic exercise changes in MT in a PPR were reported, but no direct relation to the increase in blood pressure increase was established.[50–52] During exhaustive dynamic exercise blood flow may decline due to hypocapnia, because in both retina and choroid the level of perfusion is strongly dependent on pCO_2_ levels.[53–58] The technique was also used to study dynamic autoregulation after inducing systemic hypotension by the tigh-cuff technique.[59] Generally it needs to be considered that in the choroid the blood flow response to changes in blood pressure may strongly depend on the way blood pressure is modified, because of the rich neuronal innervation of blood vessels and the sympathetic and parasympathetic input.[60–62]

LSFG was previously employed to study autoregulation in several animal studies. ONH blood flow autoregulation was investigated in rabbits[63, 64] during an artificial increase in IOP. In non-human primates static[65–67] and dynamic autoregulation [68, 69] were studied. ONH blood flow regulation was shown to decline in parallel with neural degeneration induced by ocular hypertension due to an unknown mechanism.[70] As compared to human studies such experiments have the advantage that blood pressure can be more easily controlled, but have the disadvantage that anesthesia may alter the autoregulatory response to an unknown degree.

The data of the present study may also be relevant for validation of blood flow measurements based on either Doppler optical coherence tomography (OCT) or OCT angiography. In larger vessels techniques have been developed to study retinal blood flow based on either double circular scans around the ONH,[71, 72] multi-beam approaches,[73–78] 3-D datasets[79] or enface OCT images[80] and some of these approaches have also been validated against invasive microsphere technology.[81] In the microvasculature some attempts have been made to extract quantitative data from OCT angiograms,[82, 83] but none of these techniques is adequately validated. In order to relate the complex OCT signal to blood flow or blood velocity in the microvasculature, all the issues mentioned above for LSFG must be taken into account. In addition, light scattering has to be considered for short-coherence light as used for OCT applications.

Some limitations of the present study need to be considered. Human validation experiments using LSFG were so far only done in Japanese populations. As such experience with the system in subjects of European descent is very limited to date.[25, 84] Inter-race difference in the LSFG signal may, however, well be expected because of differences in fundus pigmentation. Another limitation is related to the fact that MT and MAP were only measured every minute in the present experiments. In an elegant recent experiment Chiquet and co-workers[18] were capable of continuously measuring blood flow and blood pressure, but this is technically not possible with the commercial LSFG device. Finally, IOP was only measured at baseline and not during isometric exercise. We have, however, previously shown that this limitation is small when OPP is calculated.[85]

In conclusion we present data on the response of MT signal in the ONH and PPR during isometric exercise using LSFG. Data in the ONH resemble what has previously been shown using LDF, although values obtained for upper limit of autoregulation are slightly lower. In the PPR both retinal and choroidal circulation may contribute to the signal, but the relative ratio is unknown. LSFG is a clinically applicable tool to study ONH autoregulation in humans.

## Supporting information

S1 Data Set This file contains the data underlying the findings of this study. (XLSX)

## References

[1] Hayreh SS. The blood supply of the optic nerve head and the evaluation of it—myth and reality. Prog Retin Eye Res. 2001;20(5):563–93. Epub 2001/07/27. . 11470451 10.1016/s1350-9462(01)00004-0

[2] Schmetterer L. Ocular perfusion abnormalities in glaucoma. Part 1. Anatomy and physiology, measurement of blood flow. Russ Ophthalmol J. 2015;3:100–9.

[3] Flammer J, Orgul S, Costa VP, Orzalesi N, Krieglstein GK, Serra LM, et al The impact of ocular blood flow in glaucoma. Prog Retin Eye Res. 2002;21(4):359–93. Epub 2002/08/02. . 12150988 10.1016/s1350-9462(02)00008-3

[4] Burgoyne CF, Downs JC. Premise and prediction-how optic nerve head biomechanics underlies the susceptibility and clinical behavior of the aged optic nerve head. J Glaucoma. 2008;17(4):318–28. doi: 10.1097/IJG.0b013e31815a343b ; PubMed Central PMCID: PMC2777521. 18552618 10.1097/IJG.0b013e31815a343b PMC2777521

[5] Cherecheanu AP, Garhofer G, Schmidl D, Werkmeister R, Schmetterer L. Ocular perfusion pressure and ocular blood flow in glaucoma. Curr Opin Pharmacol. 2013;13(1):36–42. doi: 10.1016/j.coph.2012.09.003 ; PubMed Central PMCID: PMCPMC3553552. 23009741 10.1016/j.coph.2012.09.003 PMC3553552

[6] Flammer J, Mozaffarieh M. Autoregulation, a balancing act between supply and demand. Can J Ophthalmol. 2008;43(3):317–21. Epub 2008/05/22. doi: 10.3129/i08-056 . 18493273 10.3129/i08-056

[7] Schmidl D, Garhofer G, Schmetterer L. The complex interaction between ocular perfusion pressure and ocular blood flow—relevance for glaucoma. Exp Eye Res. 2011;93(2):141–55. doi: 10.1016/j.exer.2010.09.002 . 20868686 10.1016/j.exer.2010.09.002

[8] Schmetterer L, Garhofer G. How can blood flow be measured? Surv Ophthalmol. 2007;52 Suppl 2:S134–8. Epub 2007/12/06. doi: 10.1016/j.survophthal.2007.08.008 . 17998038 10.1016/j.survophthal.2007.08.008

[9] Riva CE, Titze P, Hero M, Movaffaghy A, Petrig BL. Choroidal blood flow during isometric exercises. Invest Ophthalmol Vis Sci. 1997;38(11):2338–43. Epub 1997/10/31. . 9344357

[10] Pillunat LE, Anderson DR, Knighton RW, Joos KM, Feuer WJ. Autoregulation of human optic nerve head circulation in response to increased intraocular pressure. Exp Eye Res. 1997;64(5):737–44. Epub 1997/05/01. doi: 10.1006/exer.1996.0263 . 9245904 10.1006/exer.1996.0263

[11] Movaffaghy A, Chamot SR, Petrig BL, Riva CE. Blood flow in the human optic nerve head during isometric exercise. Exp Eye Res. 1998;67(5):561–8. Epub 1999/01/08. doi: 10.1006/exer.1998.0556 . 9878218 10.1006/exer.1998.0556

[12] Garhofer G, Resch H, Weigert G, Lung S, Simader C, Schmetterer L. Short-term increase of intraocular pressure does not alter the response of retinal and optic nerve head blood flow to flicker stimulation. Invest Ophthalmol Vis Sci. 2005;46(5):1721–5. doi: 10.1167/iovs.04-1347 . 15851574 10.1167/iovs.04-1347

[13] Schmidl D, Boltz A, Kaya S, Werkmeister R, Dragostinoff N, Lasta M, et al Comparison of choroidal and optic nerve head blood flow regulation during changes in ocular perfusion pressure. Invest Ophthalmol Vis Sci. 2012;53(8):4337–46. doi: 10.1167/iovs.11-9055 . 22661477 10.1167/iovs.11-9055

[14] Schmidl D, Boltz A, Kaya S, Palkovits S, Told R, Napora KJ, et al Role of nitric oxide in optic nerve head blood flow regulation during an experimental increase in intraocular pressure in healthy humans. Exp Eye Res. 2013;116:247–53. doi: 10.1016/j.exer.2013.09.008 . 24060346 10.1016/j.exer.2013.09.008

[15] Schmidl D, Boltz A, Kaya S, Lasta M, Pemp B, Fuchsjager-Mayrl G, et al Role of nitric oxide in optic nerve head blood flow regulation during isometric exercise in healthy humans. Invest Ophthalmol Vis Sci. 2013;54(3):1964–70. doi: 10.1167/iovs.12-11406 . 23439596 10.1167/iovs.12-11406

[16] Boltz A, Told R, Napora KJ, Palkovits S, Werkmeister RM, Schmidl D, et al Optic nerve head blood flow autoregulation during changes in arterial blood pressure in healthy young subjects. PLoS One. 2013;8(12):e82351 doi: 10.1371/journal.pone.0082351 ; PubMed Central PMCID: PMCPMC3855769. 24324774 10.1371/journal.pone.0082351 PMC3855769

[17] Boltz A, Schmidl D, Werkmeister RM, Lasta M, Kaya S, Palkovits S, et al Regulation of optic nerve head blood flow during combined changes in intraocular pressure and arterial blood pressure. J Cereb Blood Flow Metab. 2013;33(12):1850–6. doi: 10.1038/jcbfm.2013.137 ; PubMed Central PMCID: PMCPMC3851895. 23921903 10.1038/jcbfm.2013.137 PMC3851895

[18] Chiquet C, Lacharme T, Riva C, Almanjoumi A, Aptel F, Khayi H, et al Continuous response of optic nerve head blood flow to increase of arterial blood pressure in humans. Invest Ophthalmol Vis Sci. 2014;55(1):485–91. Epub 2013/12/21. doi: 10.1167/iovs.13-12975 . 24355824 10.1167/iovs.13-12975

[19] Takayama J, Tomidokoro A, Ishii K, Tamaki Y, Fukaya Y, Hosokawa T, et al Time course of the change in optic nerve head circulation after an acute increase in intraocular pressure. Invest Ophthalmol Vis Sci. 2003;44(9):3977–85. Epub 2003/08/27. . 12939318 10.1167/iovs.03-0024

[20] Robinson F, Riva CE, Grunwald JE, Petrig BL, Sinclair SH. Retinal blood flow autoregulation in response to an acute increase in blood pressure. Invest Ophthalmol Vis Sci. 1986;27(5):722–6. Epub 1986/05/01. . 3700021

[21] Costa VP, Harris A, Anderson D, Stodtmeister R, Cremasco F, Kergoat H, et al Ocular perfusion pressure in glaucoma. Acta Ophthalmol. 2014;92(4):e252–66. doi: 10.1111/aos.12298 . 24238296 10.1111/aos.12298

[22] Sugiyama T, Araie M, Riva CE, Schmetterer L, Orgul S. Use of laser speckle flowgraphy in ocular blood flow research. Acta Ophthalmol. 2010;88(7):723–9. Epub 2009/09/04. doi: 10.1111/j.1755-3768.2009.01586.x . 19725814 10.1111/j.1755-3768.2009.01586.x

[23] Sugiyama T. Basic Technology and Clinical Applications of the Updated Model of Laser Speckle Flowgraphy to Ocular Diseases. Photonics. 2014;1(3):220–34.

[24] Shiga Y, Asano T, Kunikata H, Nitta F, Sato H, Nakazawa T, et al Relative flow volume, a novel blood flow index in the human retina derived from laser speckle flowgraphy. Invest Ophthalmol Vis Sci. 2014;55(6):3899–904. Epub 2014/05/31. doi: 10.1167/iovs.14-14116 . 24876283 10.1167/iovs.14-14116

[25] Luft N, Wozniak PA, Aschinger GC, Fondi K, Bata AM, Werkmeister RM, et al Measurements of Retinal Perfusion Using Laser Speckle Flowgraphy and Doppler Optical Coherence Tomography. Invest Ophthalmol Vis Sci. 2016;57(13):5417–25. doi: 10.1167/iovs.16-19896 . 27756076 10.1167/iovs.16-19896

[26] Luft N, Wozniak PA, Aschinger GC, Fondi K, Bata AM, Werkmeister RM, et al Ocular Blood Flow Measurements in Healthy White Subjects Using Laser Speckle Flowgraphy. PLoS One. 2016;11(12):e0168190 doi: 10.1371/journal.pone.0168190 . 27959905 10.1371/journal.pone.0168190 PMC5154568

[27] Sugiyama T, Utsumi T, Azuma I, Fujii H. Measurement of optic nerve head circulation: comparison of laser speckle and hydrogen clearance methods. Jpn J Ophthalmol. 1996;40(3):339–43. . 8988423

[28] Aizawa N, Yokoyama Y, Chiba N, Omodaka K, Yasuda M, Otomo T, et al Reproducibility of retinal circulation measurements obtained using laser speckle flowgraphy-NAVI in patients with glaucoma. Clin Ophthalmol. 2011;5:1171–6. Epub 2011/09/03. doi: 10.2147/OPTH.S22093 ; PubMed Central PMCID: PMCPmc3162298. 21887100 10.2147/OPTH.S22093 PMC3162298

[29] Shiga Y, Shimura M, Asano T, Tsuda S, Yokoyama Y, Aizawa N, et al The influence of posture change on ocular blood flow in normal subjects, measured by laser speckle flowgraphy. Curr Eye Res. 2013;38(6):691–8. Epub 2013/05/10. doi: 10.3109/02713683.2012.758292 . 23654357 10.3109/02713683.2012.758292

[30] Yanagida K, Iwase T, Yamamoto K, Ra E, Kaneko H, Murotani K, et al Sex-Related Differences in Ocular Blood Flow of Healthy Subjects Using Laser Speckle Flowgraphy. Invest Ophthalmol Vis Sci. 2015;56(8):4880–90. Epub 2015/08/01. doi: 10.1167/iovs.15-16567 . 26225627 10.1167/iovs.15-16567

[31] Kiss B, Dallinger S, Polak K, Findl O, Eichler HG, Schmetterer L. Ocular hemodynamics during isometric exercise. Microvasc Res. 2001;61(1):1–13. doi: 10.1006/mvre.2000.2269 . 11162191 10.1006/mvre.2000.2269

[32] Luksch A, Polska E, Imhof A, Schering J, Fuchsjager-Mayrl G, Wolzt M, et al Role of NO in choroidal blood flow regulation during isometric exercise in healthy humans. Invest Ophthalmol Vis Sci. 2003;44(2):734–9. . 12556406 10.1167/iovs.02-0177

[33] Fuchsjager-Mayrl G, Luksch A, Malec M, Polska E, Wolzt M, Schmetterer L. Role of endothelin-1 in choroidal blood flow regulation during isometric exercise in healthy humans. Invest Ophthalmol Vis Sci. 2003;44(2):728–33. . 12556405 10.1167/iovs.02-0372

[34] Briers D, Duncan DD, Hirst E, Kirkpatrick SJ, Larsson M, Steenbergen W, et al Laser speckle contrast imaging: theoretical and practical limitations. J Biomed Opt. 2013;18(6):066018 doi: 10.1117/1.JBO.18.6.066018 . 23807512 10.1117/1.JBO.18.6.066018

[35] Goodman JW. Statistical Optics. New York: Wiley & Sons; 1985.

[36] Bonner RF, Nossal R. Principles of laser Doppler flowmetry In: Shepherd AP ÖP, editor. Laser-Doppler blood flowmetry. Boston: Kluwer Academic Publishers; 1990 p. 57–72.

[37] Pournaras CJ, Rungger-Brandle E, Riva CE, Hardarson SH, Stefansson E. Regulation of retinal blood flow in health and disease. Prog Retin Eye Res. 2008;27(3):284–330. Epub 2008/05/02. doi: 10.1016/j.preteyeres.2008.02.002 . 18448380 10.1016/j.preteyeres.2008.02.002

[38] Riva CE, Geiser M, Petrig BL, Beijing 100193 PRCOBFRA. Ocular blood flow assessment using continuous laser Doppler flowmetry. Acta Ophthalmol. 2010;88(6):622–9. doi: 10.1111/j.1755-3768.2009.01621.x . 19860779 10.1111/j.1755-3768.2009.01621.x

[39] Briers JD. Statistics of fluctuation speckle patterns produced by a mixture of moving and stationary scatterers. Opt Quant Electron. 1978;10(4):364–6.

[40] Rabal HJ, Arizaga R, Cap NL, Grumel E, Trivi M. Numerical model for dynamic speckle: an approach using the movement of the scatterers. J Opt A: Pure & Appl Opt. 2003;5(5):381–5.

[41] Duncan DD, Kirkpatrick SJ. Can laser speckle flowmetry be made a quantitative tool? J Opt Soc Am A. 2008;25(1):9–15. 10.1364/josaa.25.002088 PMC2572153 18677371

[42] Takahashi H, Sugiyama T, Tokushige H, Maeno T, Nakazawa T, Ikeda T, et al Comparison of CCD-equipped laser speckle flowgraphy with hydrogen gas clearance method in the measurement of optic nerve head microcirculation in rabbits. Exp Eye Res. 2013;108:10–5. doi: 10.1016/j.exer.2012.12.003 . 23262066 10.1016/j.exer.2012.12.003

[43] Aizawa N, Nitta F, Kunikata H, Sugiyama T, Ikeda T, Araie M, et al Laser speckle and hydrogen gas clearance measurements of optic nerve circulation in albino and pigmented rabbits with or without optic disc atrophy. Invest Ophthalmol Vis Sci. 2014;55(12):7991–6. doi: 10.1167/iovs.14-15373 . 25377226 10.1167/iovs.14-15373

[44] Wang L, Cull GA, Piper C, Burgoyne CF, Fortune B. Anterior and posterior optic nerve head blood flow in nonhuman primate experimental glaucoma model measured by laser speckle imaging technique and microsphere method. Invest Ophthalmol Vis Sci. 2012;53(13):8303–9. doi: 10.1167/iovs.12-10911 ; PubMed Central PMCID: PMCPMC3525139. 23169886 10.1167/iovs.12-10911 PMC3525139

[45] Petrig BL, Riva CE, Hayreh SS. Laser Doppler flowmetry and optic nerve head blood flow. Am J Ophthalmol. 1999;127(4):413–25. Epub 1999/04/28. . 10218694 10.1016/s0002-9394(98)00437-1

[46] Schmidl D, Prinz A, Kolodjaschna J, Polska E, Luksch A, Fuchsjager-Mayrl G, et al Effect of nifedipine on choroidal blood flow regulation during isometric exercise. Invest Ophthalmol Vis Sci. 2012;53(1):374–8. doi: 10.1167/iovs.11-8536 . 22199246 10.1167/iovs.11-8536

[47] Schmidl D, Schmetterer L, Witkowska KJ, Rauch A, Werkmeister RM, Garhofer G, et al Factors associated with choroidal blood flow regulation in healthy young subjects. Invest Ophthalmol Vis Sci. 2016;(accepted for publication). 10.1167/iovs.16-20225 27787558

[48] Polska E, Luksch A, Ehrlich P, Sieder A, Schmetterer L. Measurements in the peripheral retina using LDF and laser interferometry are mainly influenced by the choroidal circulation. Curr Eye Res. 2002;24(4):318–23. Epub 2002/09/27. . 12324872 10.1076/ceyr.24.4.318.8413

[49] Kiss B, Fuchsjager G, Polak K, Findl O, Eichler HG, Schmetterer L. Age dependence of perimacular white blood cell flux during isometric exercise. Curr Eye Res. 2000;21(4):757–62. . 11120564 10.1076/ceyr.21.4.757.5549

[50] Hayashi N, Ikemura T, Someya N. Effects of dynamic exercise and its intensity on ocular blood flow in humans. Eur J Appl Physiol. 2011;111(10):2601–6. doi: 10.1007/s00421-011-1880-9 . 21373869 10.1007/s00421-011-1880-9

[51] Ikemura T, Someya N, Hayashi N. Autoregulation in the ocular and cerebral arteries during the cold pressor test and handgrip exercise. Eur J Appl Physiol. 2012;112(2):641–6. doi: 10.1007/s00421-011-2016-y . 21643919 10.1007/s00421-011-2016-y

[52] Ikemura T, Hayashi N. Ocular circulatory responses to exhaustive exercise in humans. Eur J Appl Physiol. 2012;112(9):3313–8. doi: 10.1007/s00421-012-2313-0 . 22262011 10.1007/s00421-012-2313-0

[53] Sponsel WE, DePaul KL, Zetlan SR. Retinal hemodynamic effects of carbon dioxide, hyperoxia, and mild hypoxia. Invest Ophthalmol Vis Sci. 1992;33(6):1864–9. Epub 1992/05/01. . 1582790

[54] Schmetterer L, Wolzt M, Lexer F, Alschinger C, Gouya G, Zanaschka G, et al The effect of hyperoxia and hypercapnia on fundus pulsations in the macular and optic disc region in healthy young men. Exp Eye Res. 1995;61(6):685–90. Epub 1995/12/01. . 8846840 10.1016/s0014-4835(05)80019-3

[55] Schmetterer L, Lexer F, Findl O, Graselli U, Eichler HG, Wolzt M. The effect of inhalation of different mixtures of O2 and CO2 on ocular fundus pulsations. Exp Eye Res. 1996;63(4):351–5. Epub 1996/10/01. doi: 10.1006/exer.1996.0125 . 8944542 10.1006/exer.1996.0125

[56] Luksch A, Garhofer G, Imhof A, Polak K, Polska E, Dorner GT, et al Effect of inhalation of different mixtures of O(2) and CO(2) on retinal blood flow. Br J Ophthalmol. 2002;86(10):1143–7. Epub 2002/09/18. ; PubMed Central PMCID: PMC1771321. 12234896 10.1136/bjo.86.10.1143 PMC1771321

[57] Rose K, Kulasekara SI, Hudson C. Intervisit Repeatability of Retinal Blood Oximetry and Total Retinal Blood Flow Under Varying Systemic Blood Gas Oxygen Saturations. Invest Ophthalmol Vis Sci. 2016;57(1):188–97. doi: 10.1167/iovs.15-17908 . 26795825 10.1167/iovs.15-17908

[58] Venkataraman ST, Hudson C, Fisher JA, Rodrigues L, Mardimae A, Flanagan JG. Retinal arteriolar and capillary vascular reactivity in response to isoxic hypercapnia. Exp Eye Res. 2008;87(6):535–42. Epub 2008/10/09. doi: 10.1016/j.exer.2008.08.020 . 18840429 10.1016/j.exer.2008.08.020

[59] Ikemura T, Kashima H, Yamaguchi Y, Miyaji A, Hayashi N. Inner ocular blood flow responses to an acute decrease in blood pressure in resting humans. Physiol Meas. 2015;36(2):219–30. doi: 10.1088/0967-3334/36/2/219 . 25582274 10.1088/0967-3334/36/2/219

[60] Fitzgerald ME, Tolley E, Jackson B, Zagvazdin YS, Cuthbertson SL, Hodos W, et al Anatomical and functional evidence for progressive age-related decline in parasympathetic control of choroidal blood flow in pigeons. Exp Eye Res. 2005;81(4):478–91. Epub 2005/06/07. doi: 10.1016/j.exer.2005.03.008 . 15935343 10.1016/j.exer.2005.03.008

[61] Nickla DL, Wallman J. The multifunctional choroid. Prog Retin Eye Res. 2010;29(2):144–68. doi: 10.1016/j.preteyeres.2009.12.002 ; PubMed Central PMCID: PMC2913695. 20044062 10.1016/j.preteyeres.2009.12.002 PMC2913695

[62] Li C, Fitzgerald ME, Ledoux MS, Gong S, Ryan P, Del Mar N, et al Projections from the hypothalamic paraventricular nucleus and the nucleus of the solitary tract to prechoroidal neurons in the superior salivatory nucleus: Pathways controlling rodent choroidal blood flow. Brain Res. 2010;1358:123–39. doi: 10.1016/j.brainres.2010.08.065 ; PubMed Central PMCID: PMCPMC2949519. 20801105 10.1016/j.brainres.2010.08.065 PMC2949519

[63] Shibata M, Oku H, Sugiyama T, Kobayashi T, Tsujimoto M, Okuno T, et al Disruption of gap junctions may be involved in impairment of autoregulation in optic nerve head blood flow of diabetic rabbits. Invest Ophthalmol Vis Sci. 2011;52(5):2153–9. doi: 10.1167/iovs.10-6605 . 21220555 10.1167/iovs.10-6605

[64] Shibata M, Sugiyama T, Kurimoto T, Oku H, Okuno T, Kobayashi T, et al Involvement of glial cells in the autoregulation of optic nerve head blood flow in rabbits. Invest Ophthalmol Vis Sci. 2012;53(7):3726–32. Epub 2012/05/17. doi: 10.1167/iovs.11-9316 . 22589427 10.1167/iovs.11-9316

[65] Piper C, Fortune B, Cull G, Cioffi GA, Wang L. Basal blood flow and autoregulation changes in the optic nerve of rhesus monkeys with idiopathic bilateral optic atrophy. Invest Ophthalmol Vis Sci. 2013;54(1):714–21. Epub 2013/01/05. doi: 10.1167/iovs.12-9773 ; PubMed Central PMCID: PMC3559073. 23287792 10.1167/iovs.12-9773 PMC3559073

[66] Wang L, Burgoyne CF, Cull G, Thompson S, Fortune B. Static blood flow autoregulation in the optic nerve head in normal and experimental glaucoma. Invest Ophthalmol Vis Sci. 2014;55(2):873–80. Epub 2014/01/18. doi: 10.1167/iovs.13-13716 ; PubMed Central PMCID: PMCPmc3920822. 24436190 10.1167/iovs.13-13716 PMC3920822

[67] Wang L, Cull GA, Fortune B. Optic nerve head blood flow response to reduced ocular perfusion pressure by alteration of either the blood pressure or intraocular pressure. Curr Eye Res. 2015;40(4):359–67. doi: 10.3109/02713683.2014.924146 . 24911311 10.3109/02713683.2014.924146 PMC4482253

[68] Liang Y, Fortune B, Cull G, Cioffi GA, Wang L. Quantification of dynamic blood flow autoregulation in optic nerve head of rhesus monkeys. Exp Eye Res. 2010;90(2):203–9. Epub 2009/10/27. doi: 10.1016/j.exer.2009.10.009 . 19853603 10.1016/j.exer.2009.10.009

[69] Wang L, Cull G, Burgoyne CF, Thompson S, Fortune B. Longitudinal alterations in the dynamic autoregulation of optic nerve head blood flow revealed in experimental glaucoma. Invest Ophthalmol Vis Sci. 2014;55(6):3509–16. Epub 2014/05/09. doi: 10.1167/iovs.14-14020 ; PubMed Central PMCID: PMCPmc4073995. 24812551 10.1167/iovs.14-14020 PMC4073995

[70] Cull G, Told R, Burgoyne CF, Thompson S, Fortune B, Wang L. Compromised Optic Nerve Blood Flow and Autoregulation Secondary to Neural Degeneration. Invest Ophthalmol Vis Sci. 2015;56(12):7286–92. doi: 10.1167/iovs.15-17879 ; PubMed Central PMCID: PMCPMC4642604. 26551332 10.1167/iovs.15-17879 PMC4642604

[71] Wang Y, Bower BA, Izatt JA, Tan O, Huang D. In vivo total retinal blood flow measurement by Fourier domain Doppler optical coherence tomography. J Biomed Opt. 2007;12(4):041215 Epub 2007/09/18. doi: 10.1117/1.2772871 . 17867804 10.1117/1.2772871

[72] Wang Y, Fawzi A, Tan O, Gil-Flamer J, Huang D. Retinal blood flow detection in diabetic patients by Doppler Fourier domain optical coherence tomography. Opt Express. 2009;17(5):4061–73. Epub 2009/03/05. 177000 [pii]. ; PubMed Central PMCID: PMC2821425. 19259246 10.1364/oe.17.004061 PMC2821425

[73] Werkmeister RM, Dragostinoff N, Palkovits S, Told R, Boltz A, Leitgeb RA, et al Measurement of absolute blood flow velocity and blood flow in the human retina by dual-beam bidirectional Doppler fourier-domain optical coherence tomography. Invest Ophthalmol Vis Sci. 2012;53(10):6062–71. Epub 2012/08/16. doi: 10.1167/iovs.12-9514 . 22893675 10.1167/iovs.12-9514

[74] Werkmeister RM, Palkovits S, Told R, Groschl M, Leitgeb RA, Garhofer G, et al Response of retinal blood flow to systemic hyperoxia as measured with dual-beam bidirectional Doppler Fourier-domain optical coherence tomography. PLoS One. 2012;7(9):e45876 Epub 2012/10/03. doi: 10.1371/journal.pone.0045876 ; PubMed Central PMCID: PMC3445512. 23029289 10.1371/journal.pone.0045876 PMC3445512

[75] Dai C, Liu X, Zhang HF, Puliafito CA, Jiao S. Absolute retinal blood flow measurement with a dual-beam Doppler optical coherence tomography. Invest Ophthalmol Vis Sci. 2013;54(13):7998–8003. Epub 2013/11/14. doi: 10.1167/iovs.13-12318 ; PubMed Central PMCID: PMC3858018. 24222303 10.1167/iovs.13-12318 PMC3858018

[76] Trasischker W, Werkmeister RM, Zotter S, Baumann B, Torzicky T, Pircher M, et al In vitro and in vivo three-dimensional velocity vector measurement by three-beam spectral-domain Doppler optical coherence tomography. J Biomed Opt. 2013;18(11):116010 Epub 2013/11/20. doi: 10.1117/1.JBO.18.11.116010 . 24247747 10.1117/1.JBO.18.11.116010

[77] Doblhoff-Dier V, Schmetterer L, Vilser W, Garhofer G, Groschl M, Leitgeb RA, et al Measurement of the total retinal blood flow using dual beam Fourier-domain Doppler optical coherence tomography with orthogonal detection planes. Biomed Opt Express. 2014;5(2):630–42. doi: 10.1364/BOE.5.000630 ; PubMed Central PMCID: PMC3920891. 24575355 10.1364/BOE.5.000630 PMC3920891

[78] Haindl R, Trasischker W, Wartak A, Baumann B, Pircher M, Hitzenberger CK. Total retinal blood flow measurement by three beam Doppler optical coherence tomography. Biomed Opt Express. 2016;7(2):287–301. doi: 10.1364/BOE.7.000287 ; PubMed Central PMCID: PMCPMC4771449. 26977340 10.1364/BOE.7.000287 PMC4771449

[79] Baumann B, Potsaid B, Kraus MF, Liu JJ, Huang D, Hornegger J, et al Total retinal blood flow measurement with ultrahigh speed swept source/Fourier domain OCT. Biomed Opt Express. 2011;2(6):1539–52. Epub 2011/06/24. doi: 10.1364/BOE.2.001539 ; PubMed Central PMCID: PMC3114222. 21698017 10.1364/BOE.2.001539 PMC3114222

[80] Lee B, Choi W, Liu JJ, Lu CD, Schuman JS, Wollstein G, et al Cardiac-Gated En Face Doppler Measurement of Retinal Blood Flow Using Swept-Source Optical Coherence Tomography at 100,000 Axial Scans per Second. Invest Ophthalmol Vis Sci. 2015;56(4):2522–30. doi: 10.1167/iovs.14-16119 ; PubMed Central PMCID: PMCPMC4416527. 25744974 10.1167/iovs.14-16119 PMC4416527

[81] Told R, Wang L, Cull G, Thompson SJ, Burgoyne CF, Aschinger GC, et al Total Retinal Blood Flow in a Nonhuman Primate Optic Nerve Transection Model Using Dual-Beam Bidirectional Doppler FD-OCT and Microsphere Method. Invest Ophthalmol Vis Sci. 2016;57(3):1432–40. doi: 10.1167/iovs.16-19140 . 27031838 10.1167/iovs.16-19140

[82] Jia Y, Morrison JC, Tokayer J, Tan O, Lombardi L, Baumann B, et al Quantitative OCT angiography of optic nerve head blood flow. Biomed Opt Express. 2012;3(12):3127–37. doi: 10.1364/BOE.3.003127 ; PubMed Central PMCID: PMCPMC3521313. 23243564 10.1364/BOE.3.003127 PMC3521313

[83] Zhi Z, Cepurna WO, Johnson EC, Morrison JC, Wang RK. Impact of intraocular pressure on changes of blood flow in the retina, choroid, and optic nerve head in rats investigated by optical microangiography. Biomed Opt Express. 2012;3(9):2220–33. Epub 2012/10/02. doi: 10.1364/BOE.3.002220 ; PubMed Central PMCID: PMC3447563. 23024915 10.1364/BOE.3.002220 PMC3447563

[84] Wozniak PA, Luft N, Aschinger G, Fondi K, Bata AM, Witkowska KJ, et al The assessment of ocular blood flow with laser speckle flowgraphy in healthy caucasian. Acta Ophthalmol. 2016;94: doi: 10.1111/j.755-3768.2016.0391

[85] Boltz A, Schmidl D, Weigert G, Lasta M, Pemp B, Resch H, et al Effect of latanoprost on choroidal blood flow regulation in healthy subjects. Invest Ophthalmol Vis Sci. 2011;52(7):4410–5. doi: 10.1167/iovs.11-7263 . 21498617 10.1167/iovs.11-7263

